# Biallelic germline *DDX41* variants in a patient with bone dysplasia, ichthyosis, and dysmorphic features

**DOI:** 10.1007/s00439-024-02708-8

**Published:** 2024-10-25

**Authors:** Prashant Sharma, Jason R. McFadden, F. Graeme Frost, Thomas C. Markello, Dorothy K. Grange, Wendy J. Introne, William A. Gahl, May Christine V. Malicdan

**Affiliations:** 1 NIH Undiagnosed Diseases Program, Common Fund, National Human Genome Research Institute, National Institutes of Health, Bethesda, MD 20892 USA; 2 Medical Genetics Branch, National Human Genome Research Institute, National Institutes of Health, Bethesda, MD 20892 USA; 3 Department of Pediatrics, Washington University School of Medicine, St. Louis, MO 63110 USA

## Abstract

DDX41 (DEAD‑box helicase 41) is a member of the largest family of RNA helicases. The DEAD-box RNA helicases share a highly conserved core structure and regulate all aspects of RNA metabolism. The functional role of DDX41 in innate immunity is also highly conserved. DDX41 acts as a sensor of viral DNA and activates the STING-TBK1-IRF3-type I IFN signaling pathway. Germline heterozygous variants in *DDX41* have been reported in familial myelodysplasia syndrome (MDS)/acute myeloid leukemia (AML) patients; most patients also acquired a somatic variant in the second *DDX41* allele. Here, we report a patient who inherited compound heterozygous *DDX41* variants and presented with bone dysplasia, ichthyosis, and dysmorphic features. Functional analyses of the patient-derived dermal fibroblasts revealed a reduced abundance of DDX41 and abrogated activation of the IFN genes through the STING-type I interferon pathway. Genome-wide transcriptome analyses in the patient’s fibroblasts revealed significant gene dysregulation and changes in the RNA splicing events. The patient’s fibroblasts also displayed upregulation of periostin mRNA expression. Using an RNA binding protein assay, we identified DDX41 as a novel regulator of periostin expression. Our results suggest that functional impairment of DDX41, along with dysregulated periostin expression, likely contributes to this patient’s multisystem disorder.

**Supplementary Information:**

The online version contains supplementary material available at 10.1007/s00439-024-02708-8.

## Introduction

DEAD‑box helicase 41 (DDX41) is part of a ubiquitous class of RNA helicases that unwind double-stranded RNA structures and remodel RNA-protein complexes in an energy‑dependent manner via nucleotide triphosphate hydrolysis (Tanner and Linder 2001). The core of DDX41 is organized into two conserved domains: the DEAD domain (Domain I, containing the conserved amino acid sequence Asp-Glu-Ala-Asp) and a Helicase domain (Domain II). These two domains include a series of characteristic motifs involved in nucleotide binding, RNA binding, and ATP hydrolysis (Jiang et al. 2017). The N-terminal region harbors a nuclear localization signal (NLS) and a coiled-coil structure (aa88-120) (Abdul-Ghani et al. 2005; Jiang et al. 2017; Ma et al. 2018).

The functional role of DDX41 in the innate immune response is highly conserved (Zhang et al. 2011). DDX41 is a sensor for cytosolic double-stranded DNA (dsDNA) and cyclic dinucleotides, such as di‑GMP and di‑AMP. Its interaction with the signaling adaptor Stimulator of Interferon Genes (STING) primarily relies on its DEAD domain. Activation of STING initiates a cascade involving the recruitment of TANK-binding kinase 1 (TBK1) and the transcription factor interferon response factor 3 (IRF3), ultimately leading to the production of type I IFN (IFN-I) (Parvatiyar et al. 2012; Zhang et al. 2011). The E3-ubiquitin ligase TRIM21 interacts with DDX41 and regulates its degradation by polyubiquitination at Lys9 and Lys115 and subsequent proteasomal degradation (Zhang et al. 2013).

Loss of function *DDX41* variants have been associated with adult myelodysplastic syndrome (MDS) and/or acute myeloid leukemia (AML) (Polprasert et al. 2015). Most patients with monoallelic germline frameshift *DDX41* variants subsequently acquire a somatic variant in the other allele, resulting in biallelic *DDX41* variants that cause disease susceptibility with a median age of onset of 69 years (Kim et al. 2023; Lewinsohn et al. 2016; Polprasert et al. 2015; Sebert et al. 2019). The compromised tumor suppression observed in patients with *DDX41* variants may be related to disruption of pre-mRNA splicing, RNA processing, small nucleolar RNA processing, or ribosome biogenesis (Chlon et al. 2021; Polprasert et al. 2015), implicating the normal function of DDX41 in these crucial biological processes.

Periostin (POSTN), also commonly known as osteoblast-specific factor 2 (OSF-2), belongs to the Fasciclin family of proteins and functions as a cell adhesion molecule for preosteoblasts and is critical for their attachment to the extracellular matrix. Periostin plays a significant role in bone formation and structural stability. However, periostin’s multifaceted functions also render it influential in various disease pathologies. Elevated levels of periostin have been associated with certain types of cancers (Ratajczak-Wielgomas and Dziegiel 2015), lung diseases, such as idiopathic pulmonary fibrosis (Uchida et al. 2012) and asthma (Jia et al. 2012), and skin diseases, such as atopic dermatitis (AD) and systemic sclerosis (SSc). In AD, periostin contributes to the chronicity of inflammation (Masuoka et al. 2012), while in SSc, it plays a role in fibrosis and tissue remodeling (Yamaguchi 2014).

In this study, we identify biallelic germline variants in the *DDX41* gene in a patient with bone dysplasia, ichthyosis, and dysmorphic features. We show that biallelic variants adversely affect DDX41 protein stability, resulting in reduced signaling through the STING-TBK1 axis and dysregulation of global alternative splicing events in the patient’s fibroblasts. Remarkably, the patient’s fibroblasts exhibit an upregulation of periostin mRNA. Moreover, we identify DDX41 as a binding protein for periostin RNA, supporting its role in periostin regulation. This novel insight adds a layer of complexity to our understanding of DDX41’s multifaceted functions, emphasizing its significance in genetic disorders and the regulation of pivotal genes like periostin, thereby broadening our comprehension of its roles in human health and disease.

## Materials and methods

### Patient

The patient (proband) and family members were enrolled in the National Institutes of Health (NIH) Undiagnosed Diseases Program (UDP) (Gahl et al. 2012, 2016) under the protocol 76-HG-0238, “Diagnosis and Treatment of Patients with Inborn Errors of Metabolism and Other Genetic Disorders” approved by the Institutional Review Board (IRB) of the National Human Genome Research Institute (NHGRI). Written informed consent was obtained, and the patient was evaluated at the NIH Clinical Center.

### Whole exome and sanger sequencing

Genomic DNA from blood leukocytes was extracted following standard experimental protocols. Whole exome sequencing (WES) was performed by the NHGRI Intramural Sequencing Center (NISC). Illumina TruSeq Kit was used to prepare sequencing libraries, followed by sequencing on the Illumina HiSeq 2000. Human reference sequence (UCSC assembly hg19, NCBI build 37) was used for the reads alignment using a pipeline developed by the Undiagnosed Diseases Program (UDP). This pipeline is based on NovoAlign (Novocraft Technologies, Petaling Jaya, Malaysia) and, separately, a diploid aligner3 running on a commercial platform (Appistry Inc., St. Louis, MO). HaplotypeCaller and GenotypeGVCFs were used for variant calling, with annotations from snpEff and databases (gnomAD, ESP, and 1000 Genomes). Genotypes were called at all positions with high-quality sequence bases using the Most Probable Genotype (Teer et al. 2010) Bayesian algorithm, and variants were filtered using the graphical software tool VarSifter v1.5 (Teer et al. 2012). We focused on rare variants that fit specific genetic models and manually reviewed them in IGV, consulting databases like OMIM and PubMed for clinical data. Following ACMG-AMP guidelines, variants were evaluated for clinical gene relevance and pathogenicity. Additional criteria, such as Mendelian consistency, predicted impact, and population frequency, in combination with the gene’s biological and functional information, were given priority in cases with ambiguous clinical relevance. Validation of *DDX41* sequence variants and segregation with the disease was confirmed by Sanger sequencing. Genomic DNA flanking the site of paternal or maternal variants was amplified with HotStar Taq DNA polymerase (Qiagen, Valencia, CA) using the following primer sets: set 1 paternal, CGGCACCTTGAAGTAGGAGA and ACCTCAGGGTCTCTCCTTGG; set 2 maternal, GGATCCAATGCAGATGTGG and GGGCTCTCTTGATCCCTGTT. PCR was performed using denaturation at 95 °C for 30 s and annealing at 55 °C for 30 s (35 cycles). PCR products were purified using ExoSAP-IT (Affymetrix) and sent to the Macrogen service center (Macrogen USA, Rockville, MD) for sequencing. Sequencing files were evaluated using Sequencher v5.0 software (Gene Codes Corporation, Ann Arbor, MI).

### Cell culture, DNA treatment, and transfection

Primary dermal fibroblasts from the proband were derived from a forearm skin biopsy. Fibroblasts and human embryonic kidney (HEK)293T cells (ATCC, Manassas, VA) were cultured using the DMEM media supplemented with 10% FBS (fetal bovine serum) (Thermo Fisher Scientific, Waltman, MA), MEM nonessential amino acid solution (Sigma-Aldrich, St. Louis, MO), penicillin-streptomycin and 2 mM L-glutamine (Sigma-Aldrich). Fibroblasts from non-affected individuals (controls) were obtained from the Coriell Institute of Medical Research (GM01652, GM07522, Camden, NJ). All cell lines were grown in a 37^o^C incubator with 5.0% CO_2_. Fibroblasts were treated with 3 µg/mL Poly (dA: dT)/LyoVec (InvivoGen, San Diego, CA) for 24 h, washed twice with PBS, and processed for RNA isolation using a RNeasy kit (Qiagen) or directly lysed in 2X SDS lysis buffer (250 mM Tris-HCl [pH 6.8], 4% SDS, 10% glycerol, Complete protease and PhosSTOP phosphatase inhibitor cocktails [Roche]) for protein analysis.

For transient transfection experiments in HEK293T cells, a DDX41 plasmid (pCDNA 3.1+/DDX41-DYK) was obtained from GenScript USA (Piscataway, NJ). The patient-specific variants were generated using the Q5 Site-Directed Mutagenesis Kit (New England Biolabs, Ipswich, MA) and verified by DNA sequencing. Exponentially growing HEK293T cells were transiently transfected using Lipofectamine 3000 reagent (Thermo Fisher Scientific) according to the manufacturer’s instructions. After 48 h, cells were washed twice with PBS and lysed in 2X SDS lysis buffer for protein analysis.

### RNA extraction and quantitative PCR

Total RNA was extracted from proband and control fibroblasts using a RNeasy Mini Kit (Qiagen). Contamination of genomic DNA was removed using the Turbo DNA-free kit (Thermo Fisher Scientific) following the manufacturer’s instructions. Single-stranded cDNA was synthesized using the High-Capacity RNA-to-cDNA kit (Thermo Fisher Scientific). For relative quantification of the *DDX41* transcript levels, real-time PCR was performed with an ABI7300 Genetic Analyzer (Applied Biosystems) using the FAM-labeled Taqman Probe and primers mix (NM_016222.3; Hs00169602_m1, Thermo Fisher Scientific). The Taqman probe anneals to the boundary between exons 9 and 10 of *DDX41*. For analysis of *POSTN* transcript levels, two different FAM-labeled Taqman Probe and primers mix (NM_001135934.1; Hs01566750_m1 (exon boundary 4–5) and Hs01566734_m1(exon boundary 10–11), Thermo Fisher Scientific) were used. Reference gene RNA Polymerase II Subunit A (*POLR2A*) (NM_000937.4; Hs00172187_m1; Thermo Fisher Scientific) was used to normalize gene expression values.

### Immunoblotting

Cell lysates were prepared from 70 to 90% confluent flasks of dermal fibroblasts using RIPA lysis buffer (Sigma) supplemented with a complete protease and PhosSTOP phosphatase inhibitor cocktails (Roche) or 2X SDS lysis buffer. Cell lysis in RIPA buffer was carried out on ice for 30 min, followed by sonication, centrifugation, and transfer of supernatants to new tubes. DC Protein Assay was employed for measuring protein concentrations (Bio-Rad Laboratories, Hercules, CA). Total protein was resolved by SDS-PAGE on 4–15% Tris-Glycine Gels (Bio-Rad Laboratories, Hercules) and transferred to nitrocellulose membranes via a Trans-Blot Turbo Transfer System (Bio-Rad Laboratories) and immunoblotted as described previously (Sharma et al. 2019). Primary antibodies used were anti-DDX41 (HPA017911, Sigma), anti-FLAG M2 (F3165, Sigma) anti-periostin (AG-20B-0033-C100, AdipoGen Life Sciences), anti-TBK1 (3504T, Cell Signaling Technology), and anti-phospho-TBK1 (5483T, Cell Signaling Technology). Mouse monoclonal anti-Vinculin (V9131, Sigma-Aldrich) was used as a loading control.

### Transcriptome analysis

For transcriptome analysis and identification of differential gene expression, total RNA was extracted using Maxwell RSC simplyRNA Cells Kit (Promega, Madison, WI) from dermal fibroblasts derived from the proband and non-affected individuals (controls). Stranded poly (A) selected mRNA libraries were constructed from total RNA and were sequenced on NovaSeq 6000 S4 flow cell using version 1.5 chemistry to achieve a minimum of 50 million 150 base read pairs. The data were processed and analyzed using Partek Flow RNA-seq analysis software (Partek Incorporated, Chesterfield, MO). Briefly, RNA-seq reads were first trimmed for adapters, and then STAR aligner was used for the alignment to the human genome (hg19). The aligned reads were quantitated to the Partek E/M annotation model (hg19_gencode_19_v2) to obtain gene counts. Differentially expressed genes (DEGs) between controls and the proband were generated with DESeq2R (FDR < 0.05) using normalized counts. To evaluate the segregation of samples and consistency across replicates, a heatmap showing the expression of all differentially expressed genes was generated using Pearson correlation distances, and hierarchical clustering was done with the Ward algorithm.

To analyze alternative splicing in the transcriptome, Multivariate Analysis of Transcript Splicing (rMATS) (Shen et al. 2014) was run using the -*-variable-read-length* and *--allow-clipping flags* on the above BAM files. To evaluate the segregation of samples and consistency of splicing changes across replicates, a heatmap showing inclusion levels of all differentially spliced events was generated using Pearson correlation distances, and hierarchical clustering was performed using the Ward algorithm. To further evaluate replicate consistency and segregation, PCA was also performed on the inclusion level of all differentially spliced events.

### Immunoprecipitation (IP) of DDX41-RNA complexes from fibroblasts

RiboCluster Profiler™ RIP-Assay Kit (MBL International Corporation, Japan) was used to detect protein-RNA complexes in the fibroblasts following the manufacturer’s instructions. Briefly, total protein extracts were prepared using the lysis buffer containing protease inhibitors, RNase inhibitor, and dithiothreitol (DTT). Freshly prepared protein extracts were incubated with protein A-Sepharose beads (Roche) and rabbit polyclonal anti-DDX41 antibody or rabbit IgG overnight at 4^o^C to co-immunoprecipitate the ribonucleoprotein complexes. The ribonucleoprotein complex bound to protein A-Sepharose was washed three times, and the RNA from this ribonucleoprotein complex was isolated. Single-stranded cDNA was prepared from the RNA using the High-Capacity RNA-to-cDNA kit (Thermo Fisher Scientific), and quantitative PCR was performed using the FAM-labeled Taqman Probe and primers mix to detect the transcript levels of *p21* (positive control) and *POSTN*. For the calculation of RNA binding, 10% of immunoprecipitated ribonucleoprotein complexes were resolved by SDS-PAGE and immunoblotted with an anti-DDX41 antibody. Quantification of DDX41 bands was performed using the LI-COR ImageStudio program (LI-COR Biosciences, Lincoln, NE) and used for normalization of binding of *POSTN* RNA to DDX41 protein.

### Statistical analysis

GraphPad Prism (Boston, MA) was used for statistical analyses. A two-tailed Mann–Whitney test was employed for experiments comparing two data sets. Results were plotted as mean ± SD using GraphPad Prism software. For the determination of statistical significance, a probability (P) value of < 0.05 was considered.

## Results

### Clinical and laboratory findings

**Fig. 1:**
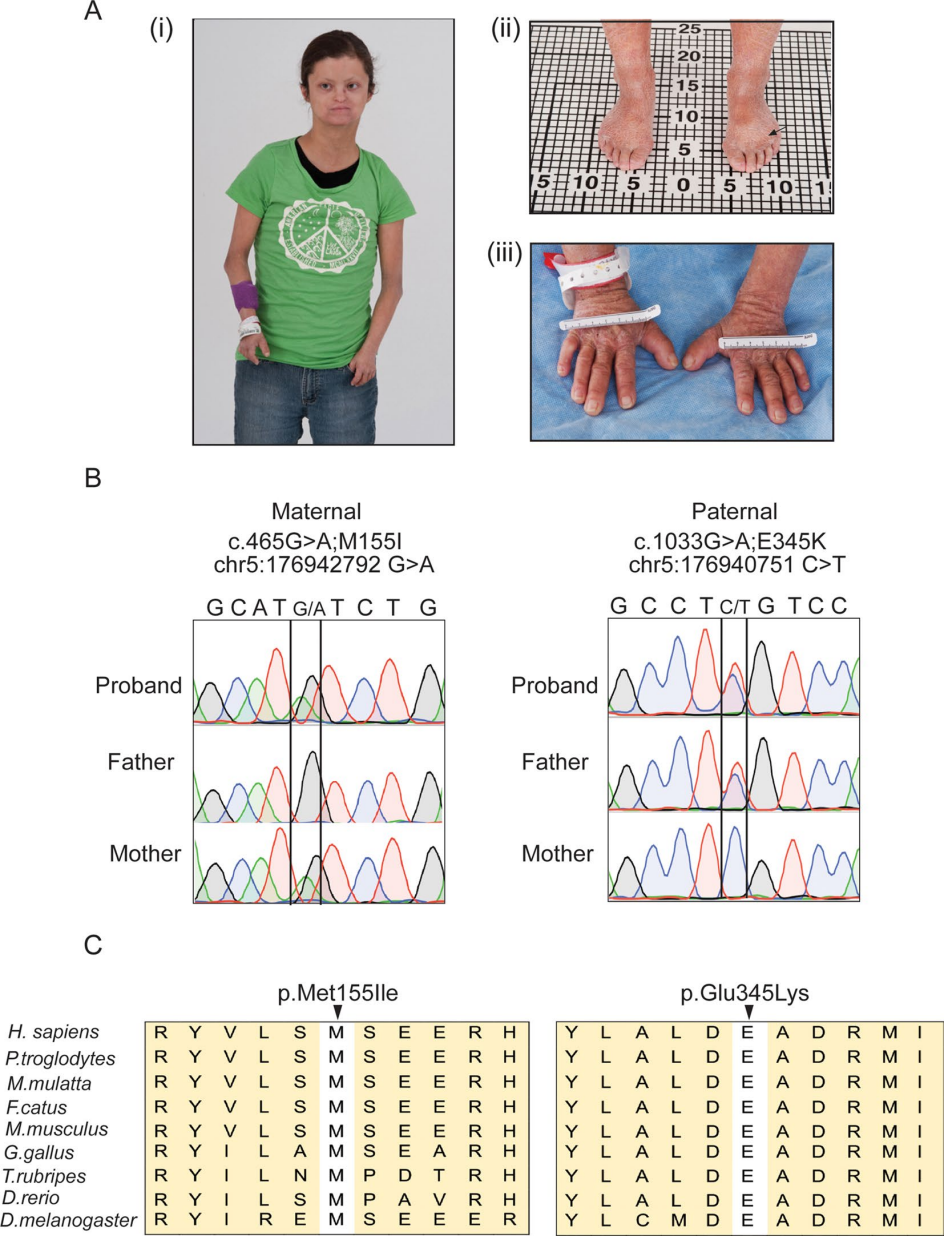
Clinical phenotype and identification of *DDX41* variants. (**A**) Photographs of the patient, taken at age 20, show dysmorphic facial features. (i-iii) The patient was diagnosed with acromesomelic dysplasia, characterized by small hands and feet. Her right upper arm, from shoulder to elbow, measures 23.5 cm, and the elbow to ulnar head length is 15.9 cm. The left upper arm is slightly longer, with the shoulder to elbow length at 24.0 cm and the elbow to ulnar head length at 16.0 cm. The total length of the right hand is 9.8 cm with a palm length of 6.0 cm, while the left hand is slightly longer with a total length of 10.0 cm and the same palm length. Her total feet measures 15.0 cm for the right foot and 14.6 cm for the left. Clinical findings include idiopathic scleroderma-like ichthyosis with skin splitting and acanthosis nigricans (arrow). (**B**) Sanger sequencing chromatograms showing biallelic variants in the proband. The proband is a compound heterozygous for NM_016222.4 (*DDX41*): c.465G > A; p. Met155Ile (inherited from the mother, left) and NM_016222.4 (*DDX41*): c.1033G > A; p. Glu345Lys (inherited from the father, right). (**C**) Alignment of missense variants in *DDX41* across multiple species including human (Homo sapiens), chimpanzee (Pan troglodytes), Rhesus macaque (Macaca mulatta), cat (Felis catus), mouse (Mus musculus), red junglefowl (Gallus gallus), Fugu rubripes (Takifugu rubripes), African clawed zebrafish (Danio rerio), and fruit fly (Drosophila melanogaster)

A 20-year-old female of non-consanguineous German and Irish descent with a complex multisystem disorder was evaluated through the National Institutes of Health’s Undiagnosed Diseases Program (see supplemental text for the complete phenotype). Her history was significant for short stature, premature closure of the epiphyses of the hands and feet, chronic ichthyotic-like skin changes, joint pain, facial dysmorphism, dental crowding, difficulty in swallowing, hyperinsulinism, and absent breast development despite otherwise normal pubertal milestones. Cognition was normal. Weight (36.7 kg, < 2.5 SD) and height (140.1 cm, < 2.5 SD); were essentially unchanged since age 14. Radiographic findings confirmed acromesomelic dysplasia, a rare progressive skeletal disorder characterized by shortening of the bones of the hands, feet, and distal limbs due to fusion of the epiphyseal growth plates (Fig. 1A). Dermatologic changes included chronic scaling, ichthyosis, predominately of the arms and legs, and acanthosis nigricans. Blood testing confirmed elevated insulin levels, but normal glycemic control.

### Exome sequencing reveals biallelic variants in *DDX41*

Whole exome sequencing followed by Sanger sequencing of genomic DNA from the proband, and her family members identified compound heterozygous variants in the *DDX41* gene; both parents were heterozygous carriers (Fig. 1B). The maternal variant (NM_016222.4: c.465G > A; p. Met155Ile) is a missense variant located toward the N-terminal while the paternal variant (NM_016222.4: c.1033G > A; p. Glu345Lys) is situated within the DEAD-box domain. Both variants affect amino acid residues (Met155 and Glu345) that are highly conserved (Fig. 1C). In addition, the Glu345 residue constitutes the binding site for cations, which is essential for ATP hydrolysis and unbinding of nucleic acid by DDX41(Singh et al. 2022; Talwar et al. 2017). The maternal variant is present in the Genome Aggregation Database (https://gnomad.broadinstitute.org/, gnomAD v2.1.1) at an extremely low total allele frequency (0.000230) only in the heterozygous state while the paternal allele was not found. The combined annotation-dependent depletion (CADD) Phred scores, which rank the deleteriousness of single nucleotide variants within the human genome, were 22.5 and 35 for the maternal and paternal variants, respectively. Both variants are predicted to be “deleterious” by MutationTester (https://mutationtaster.org/). In addition, paternal *DDX41* variant (Glu345Lys) is predicted to be “deleterious” by SIFT (https://sift.bii.a-star.edu.sg/) and “probably damaging” by PolyPhen-2 (http://genetics.bwh.harvard.edu/pph2/). Webserver wInterVar (https://wintervar.wglab.org/), which classifies genetic variants according to the ACMG/AMP 2015 guidelines (Li and Wang 2017), ranked both variants as “likely pathogenic” with re-interpreted and manually assigned evidence codes PS3, PM7, PP3 and BP1(Makishima et al. 2023) for Met155Ile variant and PS3, PM1, PP3 and BP1(Makishima et al. 2023) for Glu345Lys variant. These in silico analyses suggest that both variants are likely to affect the function of DDX41.

### Reduced DDX41 protein levels and signaling through the STING-TBK1 pathway in the proband

**Fig. 2:**
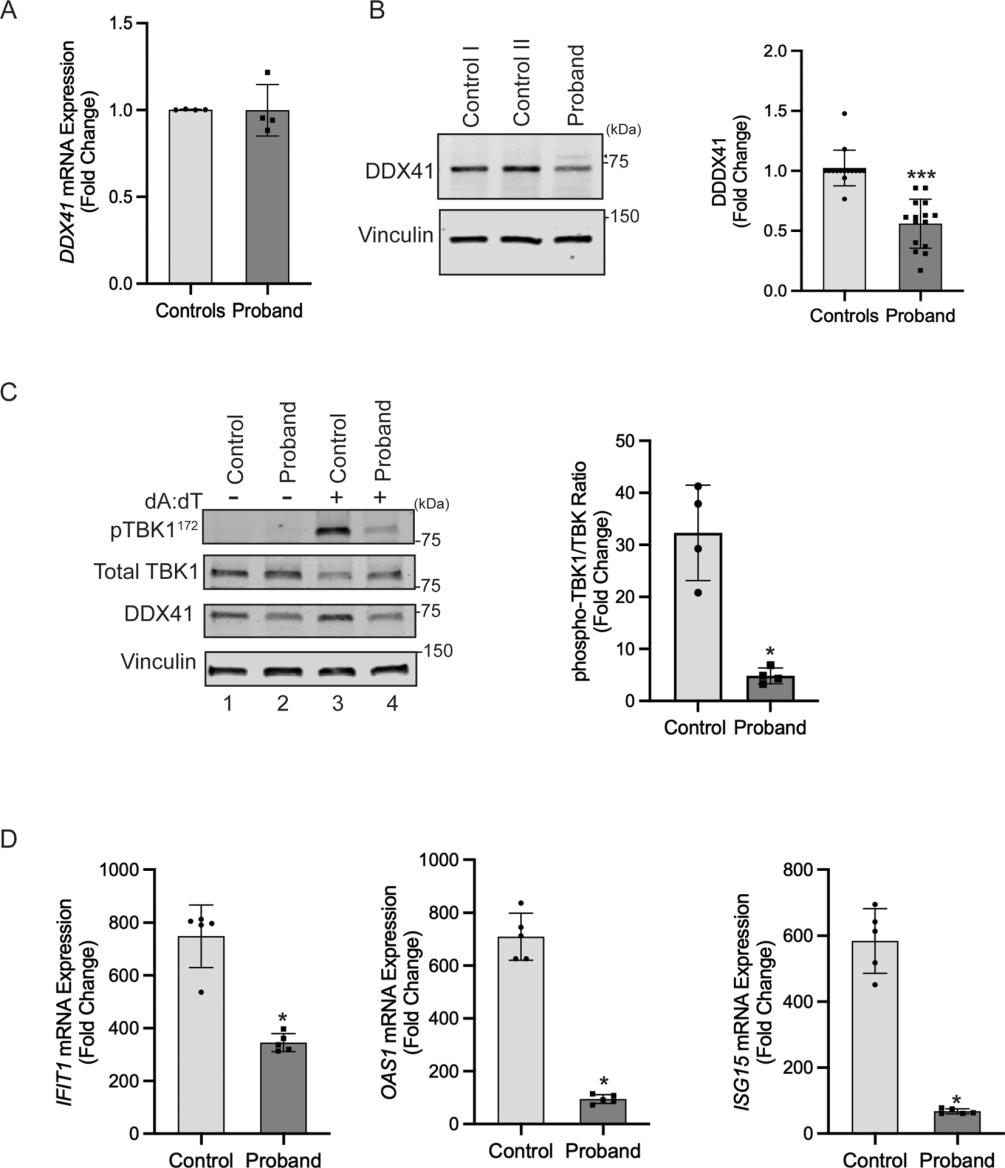
Functional analysis of *DDX41* in the proband fibroblasts. (**A**) Quantitative real-time PCR analysis showed no significant difference in mRNA expression between the between the proband and control fibroblasts (error bars indicate ± SD, *n* = 4). (**B**) Reduced abundance of DDX41 protein in the proband. (Left) Fibroblasts from controls and the proband were lysed in RIPA buffer. Levels of DDX41 were analyzed through immunoblotting of fibroblast lysates using an anti-DDX41 antibody. Immunoblotting of lysates with anti-vinculin confirmed equal loading of the samples. The positions of two molecular weight markers in kilodaltons are shown. (Right) Quantification shows fold change in protein abundance. The control value represents the mean of data from two controls (error bars indicate ± SD, *n* = 15 independent experiments, ***= *P* < 0.0001). (**C**) Reduced STING signaling in the proband. Control and the proband fibroblasts were stimulated with double-stranded DNA complex (Poly (dA: dT)/LyoVec) for 24 h and processed for protein and RNA extraction. (Left) Immunoblots of lysates with anti-phospho-TBK1, Total-TBK1, and anti-DDX41 are shown. (Right) quantitative analysis shows a significantly reduced phospho-TBK1/Total-TBK1 ratio in the proband’s fibroblasts compared to the control (error bars indicate ± SD, *n* = 4 independent experiments, *= *P* < 0.05). (**D**) Quantitative PCR analysis of cDNA shows significant downregulation of IFN response genes *IFIT1*, *OAS1*, and *ISG15* in the proband fibroblasts. Error bars indicate ± SD, *n* = 5, *= *P* < 0.05)

To investigate the effect of the identified variants on *DDX41*, we initially quantified the total mRNA levels in fibroblasts derived from the proband and unaffected controls. Quantitative polymerase chain reaction (qPCR) analysis with a Taqman probe detecting the boundary between exons 9 and 10 revealed no significant difference between levels of mRNA expression in proband and controls, suggesting that the variants do not cause transcriptional loss of *DDX41* (Fig. 2A). In contrast, direct immunoblotting of fibroblast lysates with anti-DDX41 antibody revealed significantly reduced abundance of DDX41 protein in the proband compared to control fibroblasts (Fig. 2B). Moreover, transient overexpression in HEK293T cells results in reduced steady-state levels of variants (M155I-DDX41 and E345K-DDX41) compared to wild type (WT)-DDX41 (Fig. S1). Of note, we observed an additional higher molecular weight DDX41 band in fibroblasts (Figs. 2B and 4B, arrowhead), potentially indicating a post-translationally modified form of DDX41, which was more apparent in the proband. Treatment of fibroblasts with the proteasome inhibitor MG132 led to the accumulation of DDX41 in the proband, equalizing DDX41 levels between the proband and control fibroblasts (Fig. S2). These findings suggest that diminished DDX41 levels in the proband are likely due to ubiquitination-induced proteasomal degradation.

Given the perceived role of DDX41 in regulating RNA by diverse mechanisms, we investigated if the aberrant mRNA expression of *TRIM21*, a known E3 ubiquitin ligase of DDX41(Zhang et al. 2013), is implicated in the reduced stability of DDX41 in proband. We measured *TRIM21* expression by quantitative PCR analysis and protein levels by western blotting using an anti-TRIM21 antibody. This analysis showed no significant difference in *TRIM21* mRNA and protein levels between the control and the proband fibroblasts (Fig. S3A, B). These results suggest that reduced DDX41 levels in the proband fibroblasts are likely due to the effect of biallelic variants on DDX41 protein stability.

To explore if DDX41 deficiency is associated with altered activation of the innate immune response in the proband, we evaluated the capability of the proband fibroblasts to IFN-I response upon stimulation with double-stranded DNA complex (Poly(dA: dT)/LyoVec). Poly(dA: dT) is intracellularly sensed by DDX41, leading to IFN-I production through the adaptor molecule TBK1/IRF3. Stimulation of control and proband dermal fibroblasts with Poly(dA: dT)/LyoVec for 24 h significantly increased the phosphorylation of TBK1 (Fig. 2C, top panel, lanes 1 and 3), confirming the activation of STING-TBK1 signaling. However, the proband-derived cells exhibited significantly reduced TBK1 phosphorylation compared to control (Fig. 2C, lanes 3 and 4). Moreover, transcription of the IFN-I response genes *IFIT1*, *OAS2*, and *ISG15* was significantly downregulated in proband-derived dermal fibroblasts (Fig. 2D). These findings indicated impaired activation of type I IFN response in the proband and suggest that decreased DDX41, resulting in reduced downstream STING-TBK1 signaling, is responsible for this effect.

### Proband fibroblasts exhibit dysregulation of gene expression and global alternative splicing

**Fig. 3:**
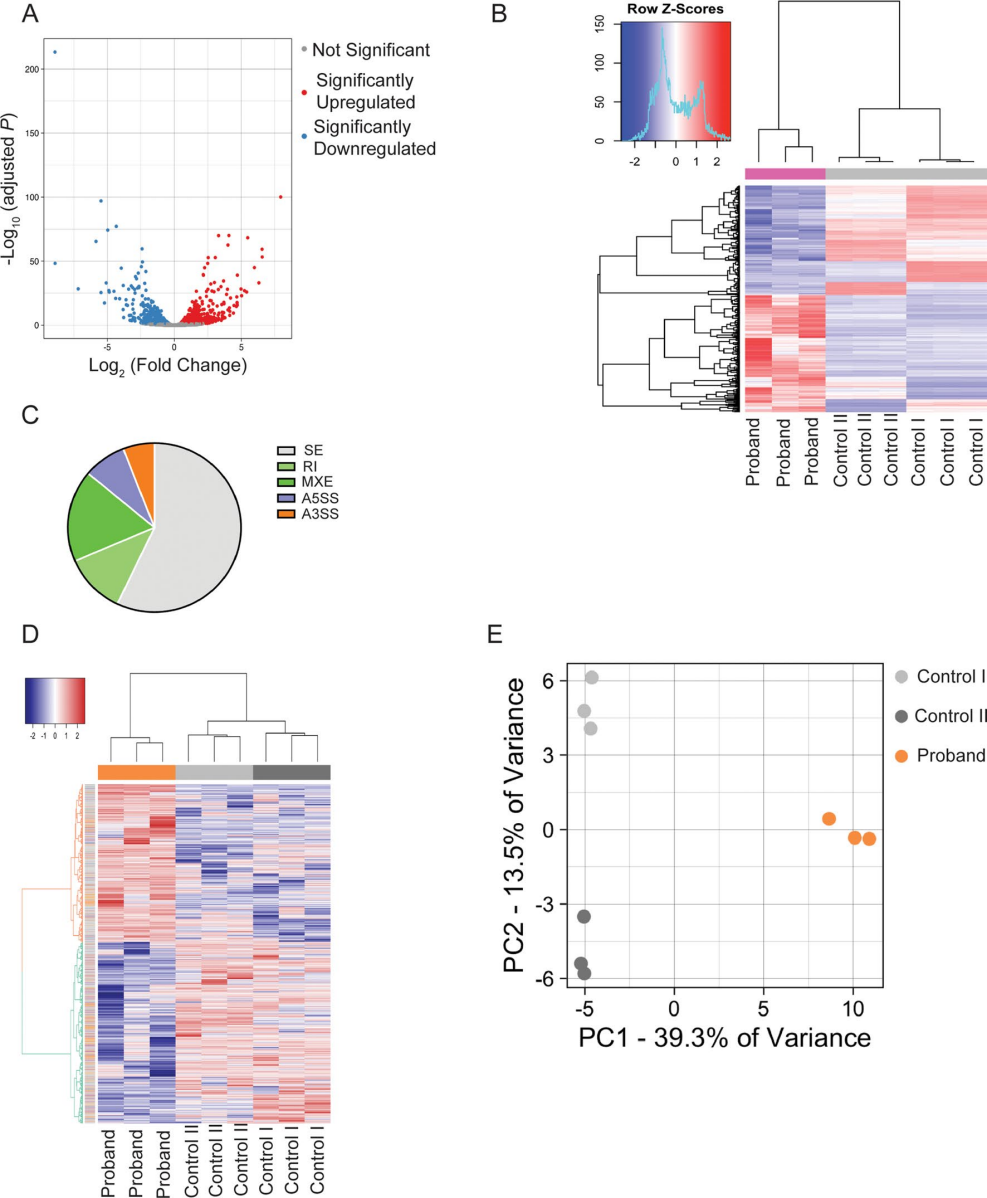
Transcriptome analysis. Analysis of paired-end RNAseq data obtained from 3 technical replicates of two controls (control I and control II) and the proband fibroblasts. In total, 65.4, 63.2, and 63.4 million aligned reads from RNA-Seq libraries made from control I, control II, and proband were obtained. The average base quality score per read (average Phred quality score) was 36.2, the average read length was 150 bp, and 100% of reads could be mapped to the human reference genome. (**A**) Volcano plot showing differentially expressed genes (DEGs) in the proband fibroblasts compared to controls. Red dots represent significantly up-regulated genes, and blue dots represent significantly down-regulated genes. (**B**) Hierarchical clustered heatmap showing the expression patterns of DEGs. Analysis of significant alternative splicing (differentially spliced) events in the proband compared to control fibroblasts using rMATs. (**C**) Distribution of 7349 RNA splicing events with significantly different frequencies between the proband and control cells: 4194 skipped exon (SE), 843 retained intron (RI), 1289 mutually exclusive exons (MXE), 590 alternative 5′ splice site (A5SS), and 433 alternative 3′ splice site (A3SS). (**D**) Hierarchical clustered heatmap showing all differentially spliced events in each sample. (**E**) Principal-component analysis (PCA) of differentially spliced event frequencies showed replicates of each sample that clustered

To comprehensively assess changes in gene expression in the proband, we performed whole-transcriptome sequencing (RNA-seq) of mRNA in dermal fibroblasts derived from the proband and two unaffected individuals (controls). Differential gene expression analysis utilizing DEseq2(R) revealed a total of 968 differentially expressed genes (DEG), including 505 significantly up-regulated and 463 significantly down-regulated genes in the proband compared to controls (Supplementary Table S1) (Fig. 3A, Volcano plot, False Discovery Rate (FDR) < 0.05). Hierarchical clustering and principal component analysis (PCA) further confirmed distinct separation between proband and control samples (Fig. 3B). Gene Ontology (GO) Biological Process (BP) term enrichment analysis of significantly enriched genes indicated dysregulation of multiple pathways, including those pathways majorly governed by alternative splicing mechanisms: angiogenesis, regulation of cell morphogenesis, and intrinsic apoptotic signaling pathway. There were also pathways related to innate immunity: leukocyte migration, interleukin-1 production, regulation of inflammatory response, response to molecules of bacterial origin, and interleukin-2 production. (Supplementary Table S2).

We also examined alternative splicing throughout the transcriptome using rMATS software (Shen et al. 2014). rMATS evaluates five types of alternative splicing events, i.e., skipped exon (SE), retained intron (RI), mutually exclusive exons (MXE), alternative 5′ splice site (A5SS), and alternative 3′ splice site (A3SS). This analysis detected 7349 differentially spliced alternative splicing events with significantly different frequencies (False Discovery Rate or FDR ≤0.05) between the proband and control cells (Fig. 3C). All samples were clustered based on differentially spliced alternative spliced events, highlighting differences between the proband and controls (Fig. 3D and E). We found that some splicing events led to differences in gene expression between the control and proband samples; the mean expression of genes with significantly different splicing events is broadly not different between controls and proband (Fig. S4). Together, these analyses showed widespread changes in gene expression and alternative splicing in proband fibroblasts, suggesting that impaired DDX41function affects different aspects of RNA biology, including RNA processing and pre-mRNA splicing. The precise mechanism requires further in-depth exploration.

### DDX41 regulates periostin

**Fig. 4:**
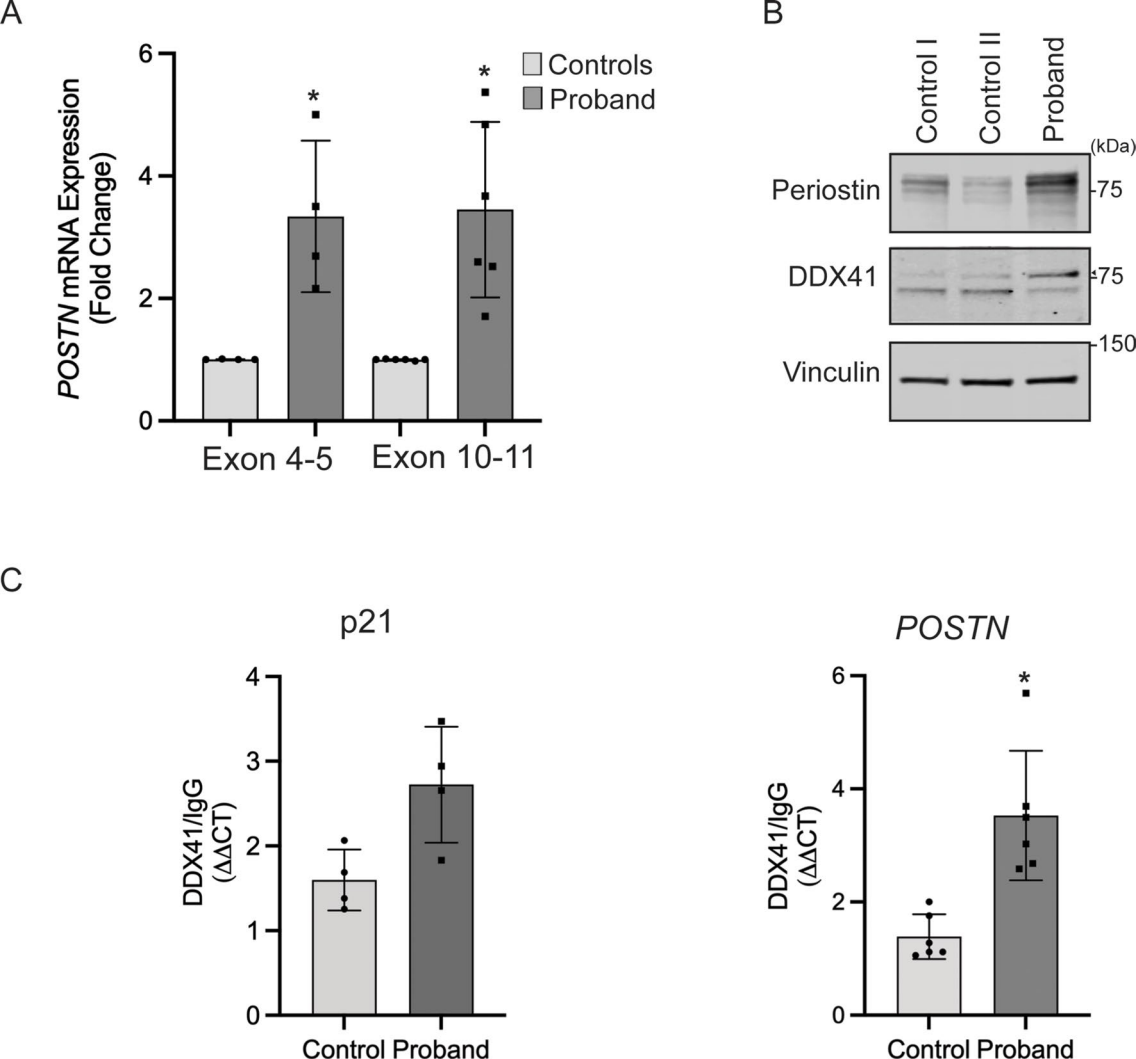
DDX41 Regulates Periostin expression. (**A**) Quantitative real-time PCR analysis of Periostin (*POSTN*) gene expression using two different Taqman Probes shows significantly increased expression of *POSTN* in the proband-derived fibroblasts compared to controls (error bars indicate ± SD, *n* = 4–5 independent experiments, *= *P* < 0.05). (**B**) (Upper panel) Levels of periostin were analyzed through immunoblotting of fibroblast lysates using an anti-periostin antibody. (Middle panel) Levels of DDX41 were analyzed using an anti-DDX41 antibody. Arrowhead indicates a post-translationally modified form of DDX41. (Lower panel) immunoblotting of lysates with anti-vinculin confirmed equal loading of the samples. The positions of molecular weight markers in kilodaltons are shown. (**C**) The binding of *p21* and *POSTN* mRNA to DDX41 protein was measured using a ribonucleoprotein immunoprecipitation assay. DDX41 was immunoprecipitated from total fibroblasts lysates with an anti-DDX41 antibody. Binding to normal rabbit IgG was used as a negative control and to calculate the relative binding of *p21* and *POSTN* mRNA to DDX41 protein. The binding of *p21* mRNA to DDX41 was used as a positive control (error bars indicate ± SD, *n* = 4–6 independent experiments, *= *P* < 0.05)

We identified Periostin (POSTN) as one of the genes highly expressed in the proband-derived dermal fibroblasts in our RNA-seq analysis. Besides the role of POSTN in bone formation, the overexpression of POSTN has been observed in different diseases characterized by fibrosis, inflammation, and tumorigenesis (Idolazzi et al. 2017). qPCR analysis confirmed significant upregulation of *POSTN* mRNA in the proband compared to controls (Fig. 4A). Consistent with increased transcription, the abundance of periostin protein was significantly higher in the proband (Fig. 4B). Analysis of periostin mRNA splicing indicated normal processing in proband-derived fibroblasts (Fig. S5).

Next, we investigated the possibility that DDX41 is directly involved in the regulation of *POSTN* RNA. We immunoprecipitated total DDX41 from the protein extracts of control and the proband-fibroblasts using anti-DDX41 antibody and analyzed the *POSTN* RNA levels by qPCR in the immunoprecipitated fraction using the ribonucleoprotein immunoprecipitation (RIP) assay. It was previously shown that DDX41 binds to the 3′UTR of *p21* RNA and acts as a repressor (Peters et al. 2017). We confirmed the validity of our assay by detecting *p21* RNA in the DDX41-immunoprecipitated fraction (Fig. 4C, Left). Importantly, we detected significant enrichment of *POSTN* RNA in an anti-DDX41 immunoprecipitated fraction compared to an IgG control. We also found that the binding of DDX41 to *POSTN* RNA was significantly elevated compared to the control (Fig. 4C, Right). These results suggest a direct role of DDX41 in regulating *POSTN* RNA.

## Discussion

DDX41 is a highly conserved protein and essential for cell viability and growth. Germline monoallelic variants in *DDX41*, often frame-shift variants in the N-terminal region (Quesada et al. 2019), have been associated with an increased risk of myeloid malignancies, including MDS and AML (Lewinsohn et al. 2016; Polprasert et al. 2015). Approximately half of MDS patients with inherited *DDX41* variants acquire a somatic variant later in life within the helicase domain, often p.R525H, in the second allele (Quesada et al. 2019). The occurrence of somatic variants is likely due to selective pressures because of the stress on hematopoiesis caused by heterozygous loss of *DDX41*.

Interestingly, germline biallelic missense *DDX41* variants have been reported in two siblings who presented with dysmorphic features and delayed psychomotor development. Both variants were identified in the DEAD domain, but only one of the two siblings developed blastic plasmacytoid dendritic cell neoplasm (BPDCN) in childhood (Diness et al. 2018). In our study, we identified a patient with biallelic missense *DDX41* variants, one from each of her unaffected parents; the maternal variant (c.465G > A; p. M155I) is in the N-terminal region, and the paternal variant (c.1033G > A; p.E345K) is within the DEAD domain. The patient was presented with multisystem congenital anomalies not previously described in *DDX41* variant carriers. A distinct mechanism of pathogenesis could likely account for differences in the clinical manifestations. We speculate that during early embryonic development, biological processes essential for proper growth and differentiation were dysregulated, resulting in a clinical manifestation at a very young age in the patient.

While *DDX41* variants are typically associated with late-onset MDS, the significance of these variants in predisposing the patient to MDS remains uncertain. Other factors, such as male predominance (92%) and the presence of additional variants, including *TP53* and *ASXL1*, increase disease susceptibility (Maciejewski et al. 2017; Polprasert et al. 2015; Quesada et al. 2019). The absence of these factors may mitigate the risk of developing MDS in our patient. Supporting this contention, an association of p.M155I germline variant has been reported in a male patient with secondary acute myeloid leukemia (sAML) (Polprasert et al. 2015). Of note, p.M155I variant was inherited from the mother without history of developing myeloid neoplasm.

Our functional analysis revealed a significant reduction in DDX41 stability in the proband’s dermal fibroblasts. Importantly, this reduction in DDX41 levels was not attributed to TRIM21, an E3-ubiquitin ligase that induces the Lys48-linked polyubiquitination and subsequent proteasome degradation of DDX41 (Zhang et al. 2013). Instead, our data suggest that the biallelic *DDX41* variants affect the stability of DDX41. This is likely due to variant-induced structural changes, resulting in DDX41 being more amenable to post-translational modification. This may ultimately lead to more efficient ubiquitination and subsequent degradation by the proteasome.

The functional role of DDX41 in the innate immune response is well established (Parvatiyar et al. 2012; Zhang et al. 2011). Knockdown of DDX41 in myeloid dendritic cells blocked the ability of cells to mount type I interferon and cytokine responses to DNA and DNA viruses (Zhang et al. 2011). DDX41 senses foreign DNA with its DEAD domain and activates STING, which translocates from the endoplasmic reticulum to the Golgi apparatus and forms a complex with TBK1. The STING-TBK1 complex activates TBK1 (phosphorylated TBK1). IRF3, upon subsequent phosphorylation and nuclear translocation, ultimately leads to the expression of type I interferons. The significant reduction in DDX41 protein levels likely accounts for the reduced signaling through the STING-TBK1 pathway. Treatment of the patient’s cells with double-stranded DNA complex results in a significantly decreased phospho-TBK1/TBK1 ratio. We used this ratio to indicate both DDX41 and downstream STING-TBK1 signaling activation. Consistent with this, the transcription of IFN-I response genes *IFIT1*, *OAS2*, and *ISG15* was significantly downregulated. These findings suggest a weakened innate immunity in response to invading microorganisms, potentially contributing to the patient’s clinical phenotype.

DEAD-box proteins, including DDX41, regulate multiple aspects of RNA biology, including RNA splicing and fine-tuning the expression of genes at various levels (Bourgeois et al. 2016; Linder and Fuller-Pace 2013). These proteins bind to RNA through conserved motifs in domains I and II (Putnam and Jankowsky 2013). The interaction of DDX41 with spliceosomal proteins, such as the major components in U2 and U5 spliceosomes has been reported (Polprasert et al. 2015), supporting a role for the DDX41 in RNA splicing. In addition, transcriptome analysis of the proband’s dermal fibroblasts revealed significant dysregulation of gene expression and disruption of alternate splicing events. GO-BP term enrichment analysis revealed significant enrichment in pathways such as “osteoblast proliferation,” “negative regulation of cell adhesion,” “regulation of inflammatory response,” “bone development”, and “response to BMP” in the patient, which may explain some of the patient’s phenotypic features. The role of BMP signaling has been described in the development of acromesomelic dysplasia (Gomez-Puerto et al. 2019). In our survey of the differentially expressed genes, we identified altered expression of genes (SMAD9 and BMPER) involved in the BMP signaling. These gene-specific alterations may explain the development of characteristic phenotypes such as acromesomelic dysplasia in the patient, while genome-wide changes indicate global deleterious consequences of DDX41 deficiency.

In our transcriptome datasets, our analysis identified upregulation of periostin (*POSTN*) in the proband’s dermal fibroblasts. POSTN, known for its role in bone remodeling and involvement in pathological processes, was thus further explored. While alternative splicing events in POSTN RNA did not explain the transcription alteration, we investigated the direct binding of *POSTN* RNA to DDX41. Our findings revealed the presence of *POSTN* RNA in DDX41 immunoprecipitants, but more importantly, we detected an increased binding of *POSTN* mRNA in the proband cells compared to the control, which could suggest a novel role of DDX41 as an RNA binding protein in regulating *POSTN* mRNA levels. Whether *POSTN* mRNA binds to DDX41 through its untranslated or coding region and the mechanism of greater binding in the proband cells requires further investigation in future studies.

In conclusion, our study provides functional insights into the consequences of *DDX41* alleles and aberrant periostin expression in our patient’s phenotype. The observed reductions in DDX41 stability, reduced IFN-I response, and transcriptome-wide RNA splicing changes highlight the intricate molecular mechanisms underlying this genetic disorder. Further research is needed to elucidate the precise details of these regulatory processes and their impact on the observed clinical features.

## Electronic supplementary material

Below is the link to the electronic supplementary material.

Supplementary Material 1

Supplementary Material 2

Supplementary Material 3

Supplementary Material 4

## Data Availability

Data is provided within the manuscript or supplementary information files.
