# Supplementary material for: Biallelic germline DDX41 variants in a patient with bone dysplasia, ichthyosis, and dysmorphic features
Source: Hum Genet. 2024 Oct 25;143(12):1445–57. doi: 10.1007/s00439-024-02708-8 (PMC11576897; doi:10.1007/s00439-024-02708-8)
Supplement: Supplementary file 1 — Supplementary Material 1 [file 439_2024_2708_MOESM1_ESM.pdf]

Biallelic germline *DDX41* variants in a patient with bone dysplasia, ichthyosis, and dysmorphic features

Human Genetics

Prashant Sharma✉ , Jason R McFadden, F Graeme Frost, Thomas C. Markello, Dorothy K. Grange, Wendy J. Introne, William A. Gahl, May Christine V. Malicdan

✉NIH Undiagnosed Diseases Program, National Human Genome Research Institute, 5625 Fishers Lane, Rockville, MD, USA. Email: sharmap@nih.gov

Clinical Report

This 20-year-old female was evaluated under clinical protocol 76-HG-0238, "Diagnosis and Treatment of Probands with Inborn Errors of Metabolism and Other Genetic Disorders", approved by the Institutional Review Board (IRB) of the National Human Genome Research Institute (NHGRI). She was enrolled in the National Institutes of Health Undiagnosed Diseases Program and provided written, informed consent.

The proband was born to non-consanguineous parents of mixed European ancestry by C-section to a 32-year-old G_7, P_3, SAB_4 mother. Birth weight was 7 lb. 2 oz. The pregnancy was complicated by occasional maternal episodes of influenza and several days of emesis following ampicillin treatment during the first trimester. At 9 months, the proband began to have recurrent middle ear infections. She was hospitalized once for otitis media, and one time for gastroenteritis and dehydration. At 14 months, she presented with floppiness, frequent choking, profuse sweating, and constipation. Urine organic acids, mucopolysaccharides, and amino acids were all normal. At 2 years, the proband was evaluated for developmental delay due to mild delays in gross motor and speech milestones, but this resolved and there were no further developmental concerns. Bone age was also noted to be delayed at 1.5 y. At 3 years, the proband was referred

to ophthalmology and diagnosed with strabismus. At 3.5 years, a dermatologist diagnosed ichthyosis vulgaris and possible atopic dermatitis.

At 6 years, the proband was referred to another dermatological consult for idiopathic scleroderma-like ichthyosis. Physical examination was significant for thick brown scales with excoriated crusts on her thighs and forearms (with a thinner component to the scales on her lower legs), as well as marked scaling of her hands and feet on the palmar and plantar surfaces and deep fissures of both heels. The differential diagnosis at the time included an eczematous eruption, in addition to ichthyosis. A skin punch biopsy of the right leg revealed orthokeratosis, follicular plugging, and hypocellular hyalinized dermal collagen. She was prescribed Triamcinolone 0.1% ointment for severely affected areas, hydrocortisone 1% ointment for mildly affected areas, Bactroban for areas of possible infection, and emollients for her heels. The proband's condition did not improve with treatment, making eczema less likely. At 9 years, she was referred to pulmonology for episodic shortness of breath, chest pains, and chest tightness, and with a family history remarkable for various collagen vascular and immune-mediated diseases. She was diagnosed with mild reactive airway disease and perennial allergic rhinitis.

At 15 years, the proband was referred to orthopedics for elbow "popping" and an unclassified skeletal dysplasia. On examination, abnormalities included small hands and feet, broadened proximal phalanxes, brachymetacarpalia, epiphyseal spines fused with the distal radius, iliac wing flare, decreased AP diameter of vertebral bodies, shortened spinal canal, mid-space hypoplasia, a small cortical lucency along the proximal medial left femur, radial heads subluxed in the elbow region, and S-shaped scoliosis of the thoracolumbar spine (with mild thoracic dextroscoliosis and mild lumbar levoscoliosis). Her skull, although prominent, was unremarkable in comparison with the facial size. Ultimately, the constellation of findings provided most

support for a diagnosis of either acrodysostosis or geleophysic dysplasia. She previously had surgery on both elbows for subluxed radial head and nerve entrapment. From a technical standpoint, the surgery was successful, but the proband's elbow popping returned after several months. She developed chronic bone and joint pain. Treatment with hydrocodone was tried but exacerbated the constipation and was discontinued.

Also at 15 years, a repeat skin punch biopsy revealed dermal fibrosis with a reduced number of eccrine units, loss of peri-adnexal fat, and minimal chronic inflammation. The constellation of histologic findings was most suggestive of longstanding morphea. She was then referred to rheumatology for non-eczematous itchy, scaly skin with concern for a scleroderma-like condition. She was prescribed minocycline but never took the drug, as her mother was concerned about dental discoloration and other side effects. The proband tried numerous creams for her scleroderma-like symptoms, but to no avail. Acanthosis nigricans on her neck and axillae were found on examination.

Genetics consultation was sought when the patient was 8 years old. Subsequent testing revealed a normal 46, XX karyotype and no mutations in the *LMNA* (Laminopathy), *ZMPSTE24, or FGFR3* genes. Very long chain fatty acids were normal. Plasma sterols were notable for borderline high 7-dehydrocholesterol and a subtle but abnormal pattern of methyl sterols, although the significance of these findings was unclear.

At 20 years, the proband was admitted to the NIH Undiagnosed Diseases Program. She presented to the NIH Clinical Center for a complex multisystem disorder that included (i) idiopathic scleroderma-like ichthyosis with skin splitting and acanthosis nigricans and (ii) abnormal skeletal growth with premature closure of the epiphyses of the hands, feet, and distal bones of the

limbs. Her clinical history was significant for short stature, irregular menstrual periods, elevated insulin levels (169 microunits/mL; normal range 2.6-25), joint pain and discomfort in the elbows and arms, stiffness in her long bones, refractive error, strabismus, crowding of teeth and cavities, absent breast development, and cyclical diarrhea and constipation. Concerns about swallowing and choking began in the first year of life; a previous esophageal manometry study reportedly showed esophageal dysmotility. The proband had normal intelligence and graduated from community college in 2010.

Physical examination revealed that the proband was ambulatory and transferred easily from bed to floor and floor to chair; she used wheelchair for her mobility because of pain in her feet with prolonged walking. She had sparse lateral eyebrows, normal anterior hairline but low posterior hairline with diffuse seborrhea. She wore glasses and has bilateral esotropia. Palpebral fissures were upslanting and eyes appear slightly proptotic. Ears measured 5.0 cm bilaterally, but earlobes were essentially absent. She had a small mid face with some facial asymmetry and a flat nasal bridge. She had normal philtrum, crowded teeth, and intact palate. Skin was notable for acanthosis nigricans in the axilla and in the antecubital fossae. There was hyperkeratosis of the elbows and the knees. There is severe dry skin and ichthyosis on the wrists, dorsum of the hands and feet, and soles the feet.

During the NIH Clinical Center admission, the proband underwent a comprehensive multidisciplinary evaluation. An endocrinology consult was performed due to the proband's abnormal bone growth and insulin resistance. Her hemoglobin A1c levels were normal (5.2%; normal range: <5.7%), as were her leptin levels (7.7 ng/mL; normal range: 0.5-15.2). Levels of anti-nuclear antibody were elevated (5.1 EU; normal range: 0.0-0.9); no additional immune function testing was performed. The proband had slightly increased testosterone levels (70.9

ng/dL; normal range: 15-70), which was thought to be related to her hyperinsulinemia (insulin serum level: 36.8 mcU/mL; normal range: 6.0-27.0). Her axial skeletal shortening was noted to "probably represent a patterning defect," rather than endocrinological dysfunction. Her glycemic control was normal, as was her fasting glucose (84 mg/dL; normal range: 70-100). Additionally, the endocrinology service noted that the proband appeared to have normally mineralized bones on plain x-ray and has appropriate bone mineral density for her size and by whole body DEXA, and attributed the absence of breast development to be a developmental issue and not hormonal as the proband had a normal gonadal axis and clinically normal labs. A consultation was done to also evaluate her swallowing, demonstrating that she had normal swallow and her difficulty chewing and swallowing could be due to her small mandible and very crowded dentition.

Gynecological consult was also sought due to the concern of absent breast development. Review of clinical history and laboratory results (thyroid stimulating hormone (TSH), prolactin (PRL), estradiol, and follicle-stimulating hormone (FSH) were all within normal limits.

A dental consult was remarkable for the findings of chronic gingivitis despite self-reported good oral hygiene. The proband reported difficulty brushing in the corner areas of her mouth and was found to have malocclusion caused by the retention of deciduous teeth. Her oral cancer exam was negative, and her lips were dry and mildly cracked but did not bleed. The proband was introduced to sulcular brushing and was recommended for extraction of teeth to release some of the crowding.

During the proband's dermatology consult, it was noted that her ichthyosis is unusual in that it predominantly involved the upper extremities and included a palmar/plantar keratoderma but largely spared the anterior and posterior trunk. Her ichthyosis did involve scaling of the perioral

area, the lips, and the scalp; however, the appearance of the ichthyosis was noted as atypical for the more common types of inherited ichthyosis (e.g., pure ichthyosis vulgaris; lamellar ichthyosis). The proband was advised to begin careful use of mild keratolytics (e.g., low concentration urea creams or Lac-Hydrin/AmLactin creams) in addition to her current regimen of baths followed by benign moisturizing creams. Histological studies of the skin biopsy on the affected area revealed no significant pathology findings except for the ichthyosis.

The proband's NIH physical therapy consult was remarkable for impairments in core muscle strength, as well as tightness throughout the lower extremities and trunk, which impaired her ability to ambulate for longer periods of times. Her gait was characterized by decreased trunk rotation, independent without assistive device, with increased hip external rotation and decreased hip and knee flexion. Measured from ASIS to the medial malleolus, her left limb measured 69 cm, while her right limb measured 68 cm. Palpable tightness was noted throughout the lower extremity musculature. All planes of cervical and lumbar active range of motion were decreased by approximately 50% from normal. Additionally, she had some scapular winging and increased upper thoracic curvature with mild scoliosis and left lower third cervical flexion, side flexion, lateral flexion, and right upper cervical lateral flexion posture. She had notable callus formation beneath the second toes bilaterally on the dorsum of the foot and the heal, and on the left lateral portion of the great toe. The proband nonetheless was independent with transfers and mobility.

The proband also had comprehensive laboratory studies, in addition to those mentioned above, which included normal complete blood count, chemistries and lipid levels, peroxisomal panel and urinalysis (detection of organic acids and amino acids, and osmolality).

Imaging studies included brain MRI, which was remarkable for a Chiari I malformation with 3.3 mm of cerebellar tonsillar protrusion; no intracranial hemorrhage was found. Her ventricles were symmetric and of normal size; no mass-effect or midline shift was seen. Bone and soft tissues were normal, as were her orbits and globes. Her paranasal sinuses and mastoid air cells were well pneumatized bilaterally. Skeletal radiological studies showed normal AP and lateral skull (no focal lesions; grossly normal skull morphology). The spine had S-shaped curvature thoracic spine with right thoracic convexity. Upper extremity had shortening of the metacarpals and digits, especially the distal phalanges; morphologic abnormality with widening or thickening of the carpal bones. Pelvis x-ray revealed small faintly sclerotic left femoral intertrochanteric lesion and slightly shortened lower legs. Review of the knee x-ray images was unremarkable.

Clinical and biochemical laboratory tests were also performed. The proband's leukocyte coenzyme Q10 level was within normal limits (84 pmol/mg protein; normal range: 43-168). A Carbohydrate Deficient Transferrin Panel for congenital disorders of glycosylation was unremarkable (i.e., provided no strong evidence that supports a congenital disorder of glycosylation). Notable findings included a significant increase of core 1 mucin Tn antigen, borderline carbohydrate transferrin levels, an elevated T/ST ratio, and normal sialyl Tn antigen levels. The proband's N-glycan structural analysis was normal. Her urinary oligosaccharide and free glycan profile was normal.

A plasma sterol- quantification by gas chromatography-mass spectrometry was performed, which was remarkable for a borderline high level of 7-dehydroxholesterol. A subtle, but abnormal, pattern of methyl sterols was also present. Her cholesterol levels were normal (156 mg/dl; normal range: < 200). It was noted, however, that the significance of this sterol pattern is

unclear. Laminopathy testing via *LMNA* gene sequencing was performed. No sequence variants in the *LMNA* gene were found.

In addition to the above comprehensive tests, DNA from the proband and her parents were sent for whole-exome sequencing on a research basis.

Family History

Paternal Family History

The patient's paternal great-grandfather died of kidney cancer that spread to the brain. The patient's paternal great-grandmother died of a stroke. The patient's paternal grandfather died of complications from stomach cancer and bone marrow problems in 1993. Her paternal grandmother is living but was diagnosed with macular degeneration in 2009. The patient's father has a clinical history significant for hypertension (for which he takes lisinopril), macular degeneration, childhood nephrotic syndrome, and glomerulonephritis.

Maternal Family History

The patient's maternal great-grandfather died of carcinoma of the brain. The patient's maternal great-grandmother died of multiple myeloma. The patient's maternal grandmother also had multiple myeloma, but it's unclear whether this was her cause of death; her maternal grandfather is living and well. The patient's mother recently died of stage IV colon cancer and has a clinical history significant for multiple sclerosis), abnormal brain MRI, and positive antinuclear antibodies (ANA) result. Across both maternal and paternal lineage, there is no family history of tuberculosis, diabetes, hemophilia, kidney disease, heart disease, or allergy.
