# Supplementary material for: Biallelic germline DDX41 variants in a patient with bone dysplasia, ichthyosis, and dysmorphic features
Source: Hum Genet. 2024 Oct 25;143(12):1445–57. doi: 10.1007/s00439-024-02708-8 (PMC11576897; doi:10.1007/s00439-024-02708-8)
Supplement: Supplementary file 2 — Supplementary Material 2 [file 439_2024_2708_MOESM2_ESM.pdf]

Biallelic germline *DDX41* variants in a patient with bone dysplasia, ichthyosis, and dysmorphic features

Human Genetics

Prashant Sharma✉, Jason R McFadden, F Graeme Frost, Thomas C. Markello, Dorothy K. Grange, Wendy J. Introne, William A. Gahl, May Christine V. Malicdan

✉NIH Undiagnosed Diseases Program, National Human Genome Research Institute, 5625 Fishers Lane, Rockville, MD, USA. Email: sharmap@nih.gov

Supplementary Figures (S1-S5)

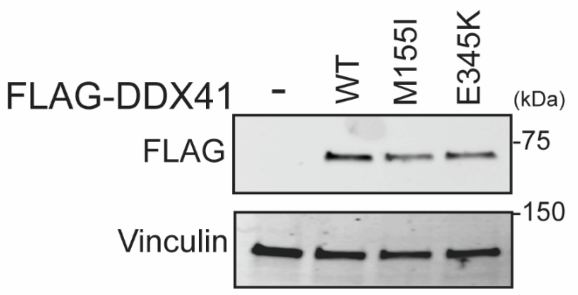

Figure S1: Reduced Protein levels of DDX41 variants in HEK 293T cells

HEK293T cells were transiently transfected with FLAG (DYK) tagged-WT-DDX41 or with variants (M155I-DDX41 and E345K-DDX41). Immunoblotting of lysates with anti-FLAG antibody revealed reduced levels of both variants compared to WT-DDX41. Anti-vinculin Immunoblotting was used to confirm equal loading of the samples.

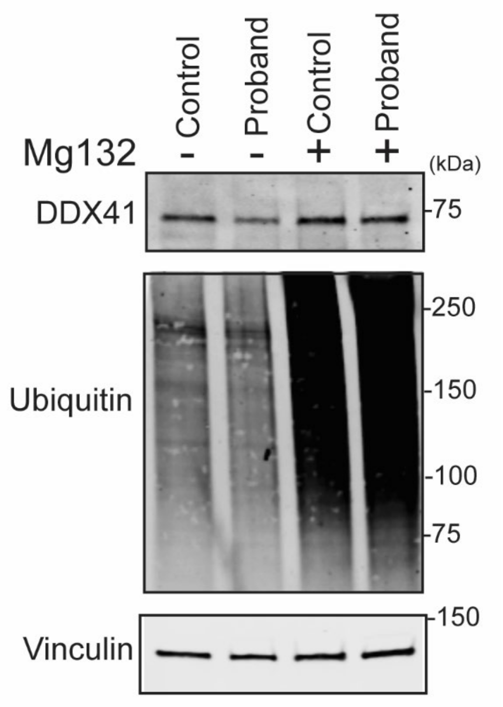

Figure S2: Proteasome inhibition restores DDX41 protein levels in the proband fibroblasts

The proteasome inhibitor MG132 treatment restored the DDX41 protein levels in the proband fibroblasts. Control and proband fibroblasts were treated with 10 μmol/L MG132 for 18h before lysis in 2X SDS buffer and subsequent immunoblotting with anti-DDX41 antibody. Anti-Ubiquitin blot shows the accumulation of ubiquitinated proteins following proteasome inhibition. Vinculin was used as a loading control.

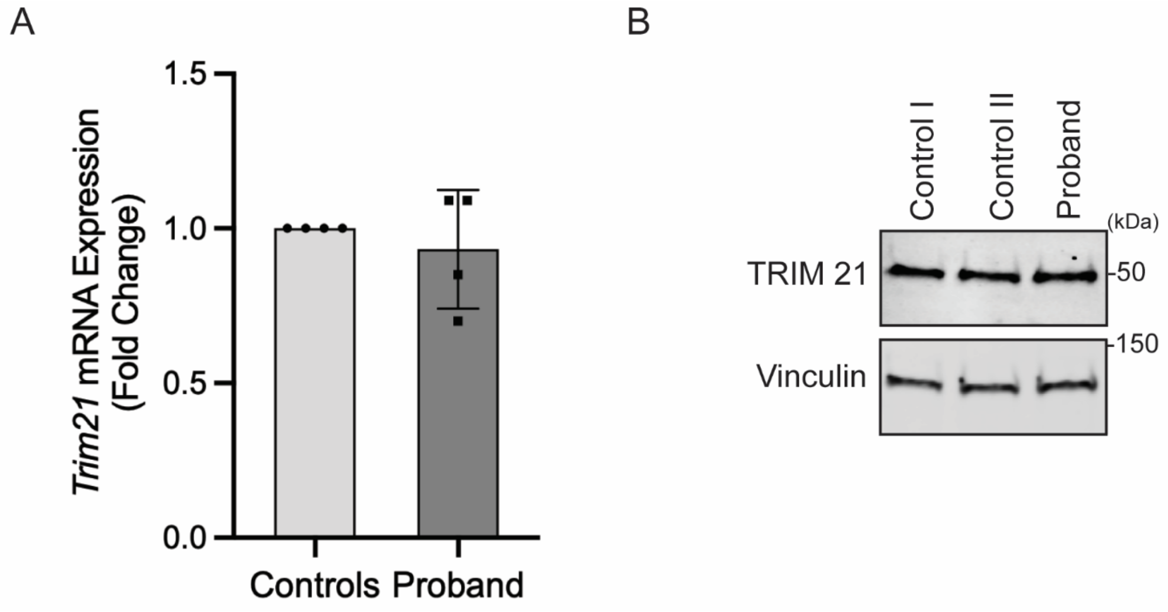

Figure S3: No Change in TRIM21 expression in the proband fibroblasts

(A) Quantitative real-time PCR analysis shows no significant difference in *TRIM21* mRNA expression between controls and proband fibroblasts (error bars indicate ± SD, n=4).

(B) Immunoblotting of the fibroblast lysates with anti-TRIM21 antibody revealed no significant difference in the TRIM21 abundance between controls and proband fibroblasts.

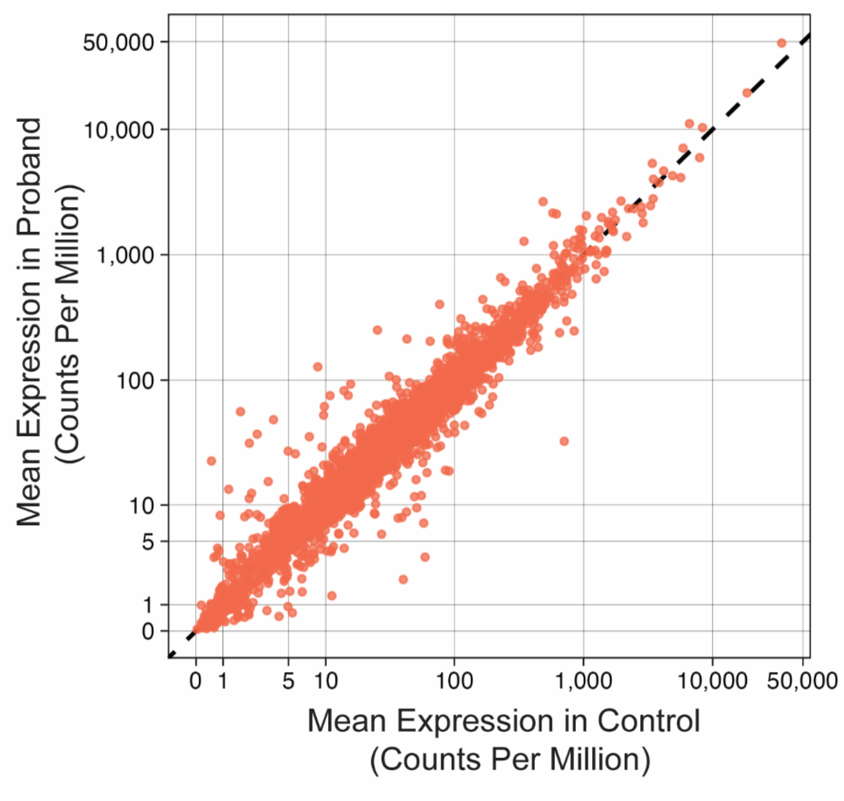

Figure S4: Correlation of Gene expression in Differentially Spliced Genes

Analysis of the mean expression of all genes with significantly altered splicing events in proband and control fibroblasts shows a consistent correlation demonstrating that splicing dysregulation has a broadly subtle if any, effect on gene expression.

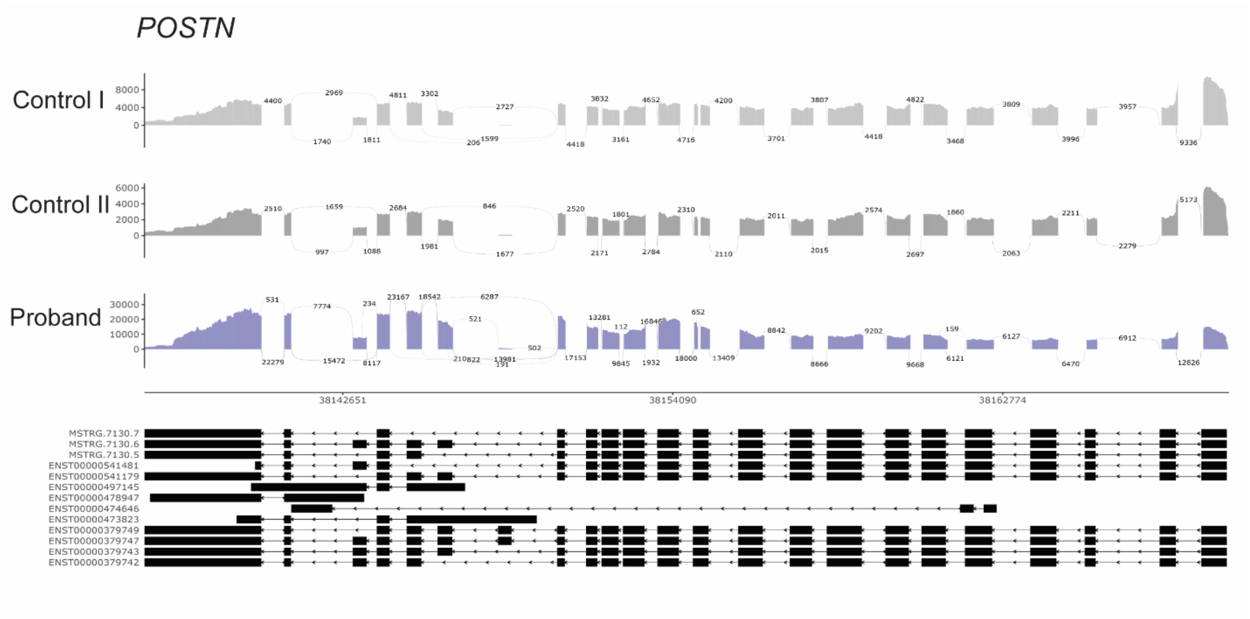

Figure S5: Sashimi plot of periostin

All reads from RNA-Seq data that mapped to the periostin (*POSTN*) gene were constructed into a Sashimi plot. Counts at each junction represent the mean of counts at that junction for all three replicates of each sample. The coordinates are in GRCH37. No significant difference in the splicing pattern between controls and proband fibroblasts.
